# The association between parity, infant gender, higher level of paternal education and preterm birth in Pakistan: a cohort study

**DOI:** 10.1186/1471-2393-11-88

**Published:** 2011-11-02

**Authors:** Kiran Shaikh, Shahirose S Premji, Marianne S Rose, Ambreen Kazi, Shaneela Khowaja, Suzanne Tough

**Affiliations:** 1 School of Nursing, Aga Khan University, Karachi, Pakistan; 2 University of Calgary, Faculty of Nursing, 2500 University Drive NW, Calgary, Alberta T2N 1N4, Canada; 3 Alberta Health Services, Calgary, Canada; 4 Department of Community Health Sciences, Aga Khan University Hospital, Stadium Road, Karachi, Pakistan; 5 Department of Pediatrics and Community Health, University of Calgary, Canada

## Abstract

**Background:**

High rates of antenatal depression and preterm birth have been reported in Pakistan. Self reported maternal stress and depression have been associated with preterm birth; however findings are inconsistent. Cortisol is a biological marker of stress and depression, and its measurement may assist in understanding the influence of self reported maternal stress and depression on preterm birth.

**Methods:**

In a prospective cohort study pregnant women between 28 to 30 weeks of gestation from the Aga Khan Hospital for Women and Children completed the A-Z Stress Scale and the Centre for Epidemiology Studies Depression Scale to assess stress and depression respectively, and had a blood cortisol level drawn. Women were followed up after delivery to determine birth outcomes. Correlation coefficients and Wilcoxon rank sum test was used to assess relationship between preterm birth, stress, depression and cortisol. Logistic regression analysis was used to determine the key factors predictive of preterm birth.

**Results:**

132 pregnant women participated of whom 125 pregnant women had both questionnaire and cortisol level data and an additional seven had questionnaire data only. Almost 20% of pregnant women (19·7%, 95% CI 13·3-27·5) experienced a high level of stress and nearly twice as many (40·9%, 95% CI 32·4-49·8%) experienced depressive symptoms. The median of cortisol level was 27·40 ug/dl (IQR 22·5-34·2). The preterm birth rate was 11·4% (95% CI 6·5-18). There was no relationship between cortisol values and stress scale or depression. There was a significant positive relationship between maternal depression and stress. Preterm birth was associated with higher parity, past delivery of a male infant, and higher levels of paternal education. Insufficient numbers of preterm births were available to warrant the development of a multivariable logistic regression model.

**Conclusions:**

Preterm birth was associated with higher parity, past delivery of a male infant, and higher levels of paternal education. There was no relationship between stress, and depression, cortisol and preterm birth. There were high rates of stress and depression among this sample suggesting that there are missed opportunities to address mental health needs in the prenatal period. Improved methods of measurement are required to better understand the psychobiological basis of preterm birth.

## Background

Preterm birth, defined as birth occurring prior to 37 completed weeks, is a worldwide health issue with a marked difference in prevalence between developed and developing countries [1-3]. The global prevalence of preterm birth is 9.6% [1]. The rate of preterm birth in Pakistan is 15·7% whereas it is 6·6% in Australia1 [4]. Preterm birth is one of the major contributors to infant mortality and morbidity [4,5]. Given the high prevalence of psychological disorder in women during pregnancy [6] it is important to understand the relationship between psychosocial risk factors and preterm birth.

Antenatal depression is common during the second and third trimesters with a systematic review showing point estimates and 95% confidence intervals of 12·8% (10·7-14·8) and 12·0% (7·4-16·7), respectively [6]. In contrast, point prevalence of 25% was identified in the third trimester of pregnant women residing in a rural sub-district of Pakistan [7]. Pakistani pregnant women may be particularly vulnerable to stress as women's health needs are not given priority [8]. Additionally, changes in family systems, specifically structures and practices [8], and values attached to birth of a male child [9] may create unique social pressures which may influence mental health. Consequently, there is a need to understand psychosocial risk and their relationships to preterm birth which may be unique for Pakistani women.

The etiologic contribution of psychosocial processes during pregnancy and preterm birth remain elusive as findings of studies examining the association between stress or depression and preterm birth have not been consistent. Although many studies demonstrate an association [10-15] others suggest that racial disparity [14,16,17] is an underlying factor. The varied concepts and models and tools used to define stress (e.g., negative life events, perceived stress, subjective feelings of anxiety, daily hassles) and depression (e.g., thought patterns, symptoms of depression), contributes to the lack of clarity about the association between psychosocial characteristics factors and preterm birth. Cortisol, which is referred to as the "stress hormone", is activated in response to stress and depression and can be measured in blood, saliva or urine [18]. Consequently, cortisol levels may be a more objective measure of stress and depression thereby facilitate our understanding of the relationship between stress, depression and preterm birth.

Stress and depression influence the hypothalamic-pituitary-adrenal (HPA) axis whereby corticotrophin-releasing-hormone (CRH) is secreted by the hypothalamus which in turn stimulates the pituitary gland to secrete adrenocorticotrophic hormone (ACTH). ACTH stimulates the adrenal cortex to secrete cortisol hormone and the adrenal medulla to secrete norepinephrine and epinephrine. Increased cortisol levels further signal the hypothalamus and pituitary gland in a negative feedback loop to decrease CRH production. However, in depressed patients the negative feedback loop malfunctions resulting in excess production of CRH; hence cortisol [19]. The increased secretion of CRH, ACTH, and cortisol stimulate prostaglandin secretion which is responsible for the contraction and dilation of the smooth muscle which may lead to preterm labor and premature rupture of membrane [15,20]. A systematic review, [18] concluded that although gestational age influenced the results, and findings were inconsistent, high level of cortisol during pregnancy was associated with preterm birth. Glynn et al. [21] found that stress in early pregnancy was related to shorter length of gestation. Women with higher cortisol levels during second trimester of pregnancy were at greater risk of preterm birth [12,13].

Studies undertaken in South Asia have examined the contribution of maternal factors like maternal education, age, parity, birth interval, and antenatal visit on preterm birth [22-24]. However, none of these studies have considered maternal stress or cortisol in relation to preterm birth. We aimed to determine the relationship between maternal stress, depression, cortisol levels, and preterm birth in pregnant women in Karachi, Pakistan. Our hypotheses were: 1) There is a positive relationship between maternal stress and depression during pregnancy and cortisol level; 2) There is a positive relationship between the cortisol level and preterm birth; 3) There is a positive relationship between maternal stress and depression during pregnancy and preterm birth; and 4) There is a positive relationship between maternal depression and stress during pregnancy.

## Methods

### Study Design

In this prospective cohort study, pregnant women (28 to 30 weeks gestation) completed the A-Z stress tool and the Centre for Epidemiology Studies Depression (CESD) scale, and provided blood sample for cortisol analysis after providing informed consent either in Urdu or English. The blood was drawn by a trained phlebotomist after the questionnaire was completed. Lab work was undertaken between 10:00 a.m. to 1:00 p.m. for consistency and in an attempt to control for diurnal variation in cortisol. Women were followed up after delivery to determine the birth outcome (preterm or term). Confidentiality and anonymity was maintained by using unique identification numbers on both the data collection tools and label for the blood sample. The study was approved by the Ethical Review Committee of the AKUH.

### Setting

Between April and May 2010 all eligible pregnant women who attended the antenatal clinics at the two centres of the Aga Khan Hospital for Women and Children (AKHWC), Kharadar and Karimabad Karachi, Pakistan were approached in the waiting area by the nurse in-charge and invited to participate in the study. Women willing to participate were referred to the researcher (KS) or a trained research assistant. The centres provide services to diverse communities which enabled recruitment of a heterogeneous sample of pregnant women. The laboratory services are provided with the collaboration of Aga Khan University Hospital (AKUH), Karachi.

### Participants: Inclusion and exclusion criteria

We enrolled all consenting pregnant women between 18 to 40 years of age who were 28 to 30 weeks gestation based on last menstrual period and planned to deliver in the same hospital. If women self-reported medical illnesses such as diabetes mellitus, thyroid disorder, chronic renal or heart disease, or uterine and cervical abnormality, or antidepressants or other psychotropic drug use during pregnancy they were excluded from the study. Women were excluded from the study if they did not deliver at the AKHWC.

### Variables of interest and their measurement

Stress was assessed with the A-Z stress tool. The A-Z stress scale which was developed by one of the researchers (AK) and includes 30 items for which responses were captured using "yes/no" format. Construct validity, test-retest reliability and inter-rater reliability, has been established for this tool. Cronbach alpha was 0·82 for the 30 items and the item-total correlations were reported to be 0·2 to 0·8, respectively [25]. The items relate to household environment (e.g., ability to make decisions), family-related concerns (e.g., relationships with husband, children, in-laws, and parents), socioeconomic concerns (e.g., access to husband's money), adverse life events, chronic illness in family, house-hold chores, and pregnancy-related concerns (e.g., unwanted pregnancy, access to health care, birth of a girl child). The two major components of the scale are the socio-environmental hassles and chronic illnesses and focus on problems specific to Pakistani pregnant women [25]. The total score guides the rater in establishing the level of stress in pregnant women. We categorized pregnant women whose scores were in the upper 75^th ^quartile and those whose scores were below the 75^th ^quartiles as having high stress levels and not high stress levels, respectively.

Depression in pregnant women was assessed by a multi-cultural validated 20-item CESD Scale. The scores can range from 0 to 60 with a cut off point of 16 and higher suggesting symptoms of depression. The scale has adequate test/retest reliability (0·51 to 0·67 over several weeks) and high internal consistency (Cronbach's alpha 0·85 to 0·90) [26].

Cortisol was measured in blood serum using the AxSYM assay system which employs Fluorescence Polarization Immunoassay (FPIA) technology to quantify the level of cortisol. The sensitivity of this system is reported to be equal to or less than 1.1 ug/dL (Abbott Laboratories, Abbott Park, IL, USA). Salivary cortisol is a non-invasive procedure and a better way to measure cortisol than other measure particularly for rapid sampling [18,27,28]; however the salivary kits and test facility is not available in Pakistan. Blood draw for cortisol was tied with other investigations whenever appropriate and feasible. Cortisol was measured as a continuous variable.

Pregnancy outcome and delivery information including gestational age at birth was obtained from the medical records and the postpartum wards of both the centres. Preterm birth was defined as birth occurring prior to 37 completed weeks while term birth was defined as birth between 37 and 42 weeks.

### Sample size

The sample size was calculated based on a secondary hypothesis related to depression, as this required the largest sample size. An 18% difference in depression among mothers who deliver at term compared to mothers who deliver preterm was reported by Field et al., [29] and at a power of 80% and alpha level of 0·05, the estimated sample size for this study was calculated at 124 pregnant women. 143 pregnant women were recruited to account for an attrition rate of 15% among women who might experience complications in pregnancy or who might not potentially deliver at the AKHWC.

### Statistical Analysis

Data were entered in EPI Info3·5·1 and then analyzed using the Statistical Package for Social Sciences (SPSS) version 16·0. Descriptive statistics were used to compare the characteristics of participants by not high stress/high stress, non-depressed and depressed and term/preterm birth status. For categorical/ordinal level variables frequencies and percentages were calculated. Fisher's exact test was used to identify group differences when comparing categorical variable. The Mann Whitney U test was used to identify group differences when comparing ordinal level data or a continuous variable when there was evidence against normality.

The Spearman's correlation coefficient was used to examine the relationship between ordinal level variables. The Wilcoxon rank sum test was used to determine the relationship between independent samples when assumptions of normality were violated. Pearson's correlation coefficient was used to determine the linear relationship between maternal stress and depression during pregnancy and preterm birth (i.e., gestational age). Logistic regression analysis was used to determine factors predictive of preterm birth. In addition to stress, depression and cortisol level, variables considered were demographics (maternal age, ethnicity, education level, education level of husband, monthly income, family system), behavioral characteristics (nature of work, hours of walking, hours of standing), and pregnancy characteristics (parity, number of children, sex of children at home, nature of present pregnancy, history of preterm birth, history of abortion, visit to antenatal clinic, hemoglobin level). Variables were considered potential predictors if p was < 0·10 for the likelihood ratio statistic from the univariable logistic model. A multivariable model was not developed as the number of preterm births was not large enough (i.e., at least 10 preterm births for every significant variable identified in the univariable logistic regression analysis) [30].

## Results

**Figure 1:**
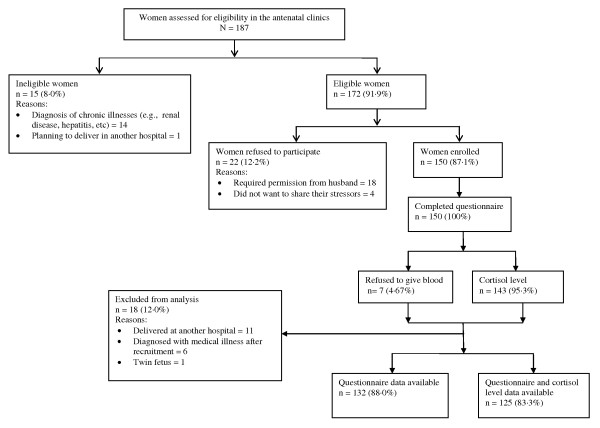
**Flow of pregnant women enrolled in the study**.

187 pregnant women were assessed for eligibility of which 172 (91·9%) were eligible. 125 pregnant women completed both the questionnaire and a cortisol level. Seven women provided only questionnaire data (n = 132). Hence, data from 125 pregnant women contributed to the analysis when relationships included cortisol, and data from 132 pregnant women contributed to the analysis when the relationship did not include cortisol (e.g., relationship between stress and depression and preterm birth, and stress and depression). Figure 1 shows the flow of mothers in the study. 26 women (19·7%, 95% CI 13·3-27·5) of the pregnant women were found to have a high level of stress and 54 (40·9%, 95% CI 32·4-49·8) reported antenatal depression. Fifteen births were preterm (11·4%, 95% CI 6·5-18·0). Tables 1 and 2 describe and compare the characteristics of mothers of term and preterm infants. Cortisol level was measured as a continuous variable; however data were not normally distributed. The median of cortisol level was 27·40 ug/dl (IQ = 22·5-34·2 ug/dl).

**Table 1:** Comparison of demographic characteristics of pregnant women by birth status.

Characteristics	Birth Status		p value
		
	Term	Preterm	
	n = 117	n = 15	
Age (year), Median	26	27	0·62†
Occupation, n (%)			0·52‡
Housewife	112 (95·7)	14 (93·3)	
Working	5 (4·3)	1 (6·6.7)	
Ethnicity, n (%)			0·82‡
Muhajir	57 (48·7)	6 (40·0)	
Memon	25 (21·4)	4 (26·7)	
Others	35 (29·9)	5 (33·3)	
Education level, n (%)			0·26†
No education	6 (5·1)	0 (0·0)	
Schooling	28 (23·9)	6 (40·0)	
Intermediate	22 (18·8)	4 (26·7)	
Graduate and above	61 (52·1)	5 (33·3)	
Education level of husband, n (%)			0·23†
No education	2 (1·7)	0 (0·0)	
Schooling	20 (17·1)	7 (46·7)	
Intermediate	23 (19·7)	0 (0·0)	
Graduate and above	72 (61·5)	8 (53·3)	
Monthly income (Rs.), n (%)			0·85†
< 10,000 (low)	*25 (23·8)	5 (33·3)	
10,001 to 40,000 (medium)	75 (71·4)	7 (46·7)	
> 40,001 (high)	5 (4·8)	3 (20·0)	
Family system, n (%)			0·76‡
Joint family system	84 (71·8)	10 (66·7)	
Nuclear family system	33 (28·2)	5 (33·3)	

> = greater than. < = less than. Rs. = Rupees. * = data missing. †Mann-Whitney U test. ‡Fisher exact test.

**Table 2:** Comparison of pregnancy characteristics by birth status.

Characteristics	Birth Status		p value
		
	Term	Preterm	
	n = 117	n = 15	
Parity, n (%)			0·11‡
Primiparous	48 (41·0)	3 (20·0)	
Multiparous	69 (59)	12 (80·0)	
Number of children, n (%)			0·10§
0	53 (45·3)	3 (20·0)	
1 to 2	51 (43·6)	10 (66·7)	
> 2	13 (11·1)	2 (13·3)	
Sex of children at home, n (%)			
Female	40 (34·2)	8 (53·3)	0.15‡
Male	44 (37·6)	11 (73·3)	**0.01‡**
Nature of present pregnancy, n (%)			0.37¶
Planned	84 (71·8)	9 (60·0)	
Unplanned	33 (28·2)	6 (40·0)	
History of preterm birth, n (%)	7 (6)	2 (13·3)	0.27¶
History of Abortion, n (%)	22 (18·8)	1 (6·7)	0.47¶
Visit to antenatal clinic, n (%)			0·89§
First trimester	70 (59·8)	9 (60·0)	
Second trimester	42 (35·9)	6 (40·0)	
Third trimester	5 (4·3)	0 (0·0)	
Hemoglobin level, n (%)			0.26§
8·1 to 9·0 mg/dl	*5 (5·2)	†0 (0·0)	
9·1 to 10·0 mg/dl	15 (15·6)	1 (7·1)	
> 10·0 mg/dl	76 (79·2)	13 (92·9)	
BMI (lb/ft^2^), Median	25·8	28·4	0.65§

> = greater than. Hb = hemoglobin. BMI = body mass index. * = 21 missing. † = 1 missing. ‡Pearson chi square. §Mann-Whitney U test. ¶Fisher exact test.

**Figure 2:**
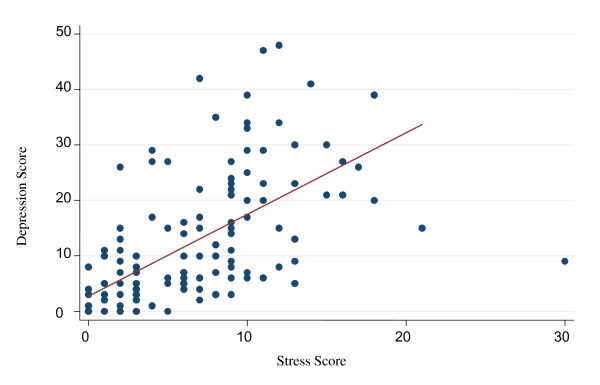
**Scatter plot showing relationship between depression and stress scores**.

There was no evidence of a positive relationship between either stress and cortisol (Spearman's rho = 0·021, *p *= 0·81) or between maternal depression and cortisol levels (rho = 0·13, *p *= 0·14). There was no significant difference (z = -0·41, *p *= 0·68) in cortisol levels of mothers with preterm infants (n = 14, median = 26.4, IQR = 23·1-33·5) and those with full term infants (n = 110, median = 27·8, IQR = 22·4-34·2). There was no significant difference between maternal stress during pregnancy and preterm birth (z = -0·69, *p *= 0·49) or between maternal depression during pregnancy and preterm birth (z = -0·70, *p *= 0·48). A significant positive relationship was identified between stress and depression as evident from the r value of 0·54 and *p *< 0·001(Figure 2).

In the univariable logistic regression analysis (Table 3) neither cortisol level (OR per 10 point increase in cortisol = 0·78, *p *= 0·507), stress (OR for high stress = 0·60, *p *= 0·519) or depression (OR for depressed = 1·4, *p *= 0·697) were associated with preterm birth. Preterm birth was associated higher parity, past delivery of a male infant, and higher levels of paternal education (Table 3).

**Table 3:** Significant predictors of preterm birth

Characteristics	Birth Status		Odds ratio	95% CI	p value*
				
	Term n = 117	Preterm n = 15			
Maternal age (years)	117	15	1.03	0.91 - 1.16	0.601
Occupation					0.692
Housewife	112	14	1.00		
Working	5	1	1.60	0.17 - 14.70	
Ethnicity					0.805
Muhajir	57	6	0.66	0.17 - 2.54	
Memon	25	4	1.00		
Others	35	5	0.89	0.22 - 3.66	
Education level					0.396
No education or some Schooling	34	6	1.00		
Intermediate and above	83	9	0.61	0.20 - 1.86	
**Education level of husband**					**0.023**
No education or some schooling	22	7	1.00		
Intermediate and above	95	8	0.26	0.087- 0.01	
Monthly Income (Rs.)					0.438
< 10,000	25	5	1.00		
≥ 10,000	80	10	0.62	0.20 - 2.00	
Family system					0.683
Joint family system	84	10	1.00		
Nuclear family system	33	5	1.27	0.40 - 4.00	
Nature of work					0.698
Sitting	7	1	1.00		
Standing	15	3	1.40	0.12 - 16.0	
Walking	30	3	0.70	0.06 - 7.78	
All	34	6	1.24	0.13 - 11.93	
Standing/walking	31	2	0.45	0.04 - 5.71	
Hours of walking					
0 to 2 hours per day	35	4	1.00		0.649
3 to 5 hours per day	69	8	1.01	0.29 - 3.60	
> 5 hours per day	13	3	1.02	0.40 - 10.3	
Hours of standing					
0 to 2 hours per day	21	3	1.00		0.539
3 to 5 hours per day	84	9	0.75	0.19 - 3.01	
> 5 hours per day	12	3	1.75	0.30 - 10.1	
**Parity**					**0.035**
Primiparous	46	2	1.00		
Multiparous	71	13	4.21	0.91 - 19.53	
Number of children					0.145
0	53	3	1.00		
1-2	51	10	3.46	0.90 - 13.31	
> 2	13	2	2.72	0.41 - 17.98	
**Sex of children at home**					
Female (no/yes)	77/40	7/8	2.20	0.74 - 6.50	0.155
**Male (no/yes)**	73/44	4/11	4.56	1.37 - 15.21	**0.008**
Nature of present pregnancy					
Planned (no/yes)	33/84	6/9	1.70	0.56 - 5.15	0.358
History of preterm birth (no/yes)	110/7	13/2	0.41	0.08 - 2.20	0.334
History of Abortion (no/yes)	95/22	14/1	3.24	0.40 - 26.0	0.198
Visit to Antenatal Clinic					0.990
First trimester	70	9	1.00		
Second/third trimester	47	6	0.99	0.33 - 2.97	
Hb Level					0.180
low	20	1	1.00		
normal	76	13	3.42	0.42 - 27.73	
Cortisol	111	14	0.98	0.91 - 1.05	0.507
Stress	117	15	0.96	0.86 - 1.08	0.519
Depression	117	15	1.01	0.96 - 1.06	0.697

CI = confidence interval. * = likelihood ratio statistic from the logistic regression model

## Discussion

Rates of stress (19·7%) and depression (40·9%) in pregnant Pakistani women in this study are high. Our findings are consistent with a rate of 39·4% among Pakistani women residing in Karachi [31] but lower than the rate of 18% reported by others [32]. Of note, this study and the study by Kazi et al., [31] used the CESD to measure symptoms of depression, which has items related to somatic symptoms of pregnancy, whereas the other Pakistani study [31] used the short form of the Aga Khan University Anxiety Depression Scale, which omits items on the somatic symptoms, which may have accounted for different findings.

The median cortisol value was 27·40 ug/dl (IQ = 22·5- 34·2 ug/dl) which is higher than values reported in the literature. For instance, Hobel et al. [13] measured serum cortisol at 28 to 30 weeks gestation and noted a mean and standard deviation of 24·74 ug/dl and 0·58 ug/dl. Ethnic differences in biological responses (i.e., HPA axis and placenta) may explain the discrepancy in the reported cortisol concentrations [33]. AKUH has a very reliable laboratory with an internal and external quality assurance program. The internal quality assurance is maintained strictly by consultants and the supervisors of the laboratory. The external quality assurance is audited by Bio-Rad EQAS (External Quality Assurance Services, USA). The quality assurance company checks the accuracy of cortisol levels every six months by verifying the cortisol level of the powdered form of 12 samples from the United States of America. In the present study, verification was requested for all cortisol levels which were reported to be high; the AKUH laboratory rechecked the analysis and indicated that all reports were "true" (i.e., there were no errors in reporting).

We identified a strong positive significant relationship between maternal stress and depression. The findings of this study therefore, support Ruiz and Avant's [20] construct that there is a positive relationship between maternal depression and stress. Studies [34-36] examining the relationship between stress and pregnancy outcomes, and/or depression, and pregnancy outcomes have asserted that there is an independent relationship to pregnancy outcomes.

We found no relationship between maternal stress and cortisol level. Ruiz and Avant's model of maternal prenatal stress [20] suggests that in pregnant women as stress increases, cortisol levels also increase, which was not identified in this study. Other studies [15,34,37] have also failed to identify a relationship between maternal stress and cortisol levels. Although findings are consistent studies used different measures of stress including the Perceived Stress Scale, [15,37] the Daily Hassles Scale, the Marital Strain Scale of Pearlin, and the Schooler Multiple scale [34], and others (e.g., negative life events, state-trait anxiety, coping style, social support and pregnancy-specific anxiety) [37].

The findings of this study may be explained by allostatic load which distinguishes between acute and chronic stress and suggests how chronic stress affects the hormonal, immune, and physiologic response in the body [38]. In situations of short term or acute stress the body regains homeostasis, whereas with exposure to long term or chronic stress the body does not regain homeostasis and allostatic load is elevated which activates the HPA and the autonomic nervous system (CRH, ACTH, cortisol, catecholamine, cytokines, etc.) to potentially increase the risk of preterm birth [39]. A study exploring a threshold model [40] suggested that stress beyond a certain level affects the relationship between stress and depression. In the present study, the A-Z stress tool measured cumulative stress and did not differentiate between acute and chronic stress which may have influenced the findings. In addition, stress, depression, and cortisol levels were measured at a single point in time, which may be inadequate for understanding the complex relationship between acute and chronic stress, depression, and cortisol levels.

We found no relationship between maternal depression and cortisol levels, which is in contrast to Ruiz and Avant's [20] model and to several studies, [29,41,42] which report higher cortisol levels in depressed women. All the studies used the CESD to measure depression, however, in this study blood samples were used to measure cortisol levels, whereas the other studies used first morning [29,41] or midmorning [42] urine samples which may influence findings [19]. A 24 hours urine collection must be obtained to acquire a more accurate measure of cortisol level as it is a "useful index of integrated 24 hours plasma free cortisol" [[19], p.46]. The role of depression as an activator of the HPA axis compared to other co-morbid factors, such as anger and anxiety, is unclear, and would perhaps influence cortisol levels [19,34]. According to Field et al. [29] co-morbid factors of depression, such as anxiety and anger, may contribute to higher cortisol levels; hence challenge the direct relationship between depression and cortisol. Future research may wish to consider the relationship between mood states as a potential influence on biology and birth outcomes.

There was no relationship between cortisol levels and preterm birth. Our findings are similar to Ruiz et al. [15] who also reported no relationship between cortisol levels and preterm birth. In both studies only a small number of women experienced preterm birth (6 out of 76 and 15 out of 132, respectively) and these small samples size increases the likelihood of making a type II error. Future research with larger numbers of preterm birth is required to better understand these complex relationships. Alternative biologic pathways also need to be considered, as cortisol may not be the most ideal biomarker of preterm birth.

Our study did not reveal any relationship between cortisol, stress and depression assessed at 28 to 30 weeks. However, studies [12,34,41,42] assessing the relationship earlier in gestation have demonstrated a relationships between some of these variables. Consequently, differences in time points of assessment may explain why our findings are inconsistent.

In our study increased parity was identified as a risk factor for preterm birth, and it has been proposed that physiologic risk factors common in multiparous women (e.g., placenta previa, placental abruption, postpartum hemorrhage) may partly explain the higher risk of preterm birth [43]. The literature is contradictory with some studies [44-46] demonstrating an association between increased parity and preterm birth while others [47,48] showing no association. A systematic review revealed no association between grand multiparity (parity 5-8) and great grand multiparity (parity > 8) and preterm birth. The role of parity may be context specific with effects of poverty combined with stress, and other factors associated with preterm birth (e.g., age, education, and ethnicity) interacting in unique ways [46,49,50] to increase risk of preterm birth in Pakistani women.

We found that preterm birth was associated with prior delivery of a male child. The proportion of male infants is higher among preterm births and this pattern is evident in different populations [51]. Pakistan has a high rate of preterm birth (15·7%) [4] and since boys are more likely to be born preterm, it is plausible that the male children at home were born preterm. The risk of preterm birth in the next pregnancy is about 15% to more than 50% [2]. Consequently, this may explain the association between male child at home and preterm birth in this study. We did not collect data on the gestational age of the male child at home, thus cannot confirm this premise.

We found that higher levels of paternal education reduced the risk of preterm birth. Education has been used as a proxy for socioeconomic status [52]. Studies from industrialized countries demonstrate an association between socioeconomic inequalities (e.g., low education level of women) and preterm birth [52-54]. In Pakistan, gender inequality in education is evident from differences in literacy rates: adult male literacy rate is 43%, whereas adult female literacy rate is 28% [55]. Our study sample was more educated than the general population with 60·5% of the husbands and 50% of the pregnant women disclosing they had a baccalaureate or higher degree. The education level of the husband may combine with other indicators of socioeconomic status (e.g., occupational status) and psychosocial factors (e.g., decision making authority of women) to reduce the risk of preterm birth or the finding may simply be due to chance.

In our study women with medical or obstetrical histories were excluded from this study, and limitations in medical records and poor health literacy of the mothers precluded classification into the other two subtypes of preterm birth - spontaneous preterm labour without premature rupture of chorioamniotic membranes (PROM), and preterm PROM with vaginal or caesarean section delivery [2,56]. Examining the subtypes of preterm birth may permit consideration of the etiological heterogeneity of preterm birth; though this view remains contentious given the similar processes leading to preterm birth [57,58]. A study [58] examining maternal risk factors in a hospital-based cohort of black and white women in relation to subtypes of preterm birth found differences in set of risk factors associated with medically indicated preterm. However, similarities were reported in set of risk factors in the other two subtypes; although differences were noted in effect size of specific risk factors. The authors concluded that grouping preterm birth into spontaneous and medically indicated preterm birth was appropriate given the current state of evidence [58]. Future studies should examine the relative contribution of each of the risk factors to preterm birth in each subtype of preterm birth.

In the present study, pregnant women were recruited from the AKHWC, which are private clinics. The sample was not completely representative of women in Karachi, Pakistan, thereby impacting the external validity of the study. Of the 187 women, 81 (54%) had an ultrasound done in the first trimester and 55 (36·7%) had an ultrasound in the second trimester; however all pregnant women had their LMP recorded in their patient chart. Although earlier ultrasound (i.e., in the first trimester) provides better pregnancy dating than ultrasound in the second trimester [59], to ensure a consistent approach in dating pregnancy, the LMP was used to determine gestational age. We measured stress, depression, and cortisol levels at a single point in time and within a two week time frame which provides a very limited assessment of the psychosocial health of the pregnant women throughout the pregnancy.

A longitudinal cohort study, with multiple measures of stress, depression, and cortisol levels, as well as a measures of anxiety and other stress hormone biomarkers may add new knowledge and enhance our understanding about the relationship between stress, depression, anxiety, cortisol levels and preterm birth. Lastly, in the present study, only 15 women experienced preterm birth which should be considered in interpreting the findings.

## Conclusion

Preterm birth was associated with higher parity, past delivery of a male infant, and higher levels of paternal education. There was no relationship between maternal stress and depression, cortisol and preterm birth when assessed cross sectionally at a single point in gestation. A larger longitudinal cohort study, with multiple measures of stress, depression, and cortisol levels, as well as a measure of anxiety may add new knowledge and enhance our understanding about the relationship between stress, depression, anxiety, cortisol levels and preterm birth. There were high rates of stress and depression among this sample suggesting that there are missed opportunities to address mental health needs in the prenatal period in Pakistan. Improved methods of measurement are required to better understand the psychobiological basis of preterm birth.

## Abbreviations

HPA: hypothalamic-pituitary-adrenal; CRH: corticotrophin-releasing-hormone; ACTH: adrenocorticotrophic hormone; AKHWC: Aga Khan Hospital for Women and Children; AKUH: Aga Khan University Hospital; SPSS: statistical package for social sciences.

## Competing interests

The authors declare that they have no competing interests.

## Authors' contributions

The work presented here was undertaken in collaboration with all authors. KS was a graduate student who undertook this work to fulfill the requirements for Masters of Science in Nursing. SP was the supervisor who was actively involved in all phases of the study including original idea, design, data analysis, interpretation and writing the report. ST, AK and SK gave ongoing feedback and suggestions during both the proposal phase and the conduct of the research. They have reviewed and edited the report. MSR guided the statistical analysis and data interpretation and contributed to the write up of this section. All authors have reviewed and approved the final version of the report.

## Pre-publication history

The pre-publication history for this paper can be accessed here:

http://www.biomedcentral.com/1471-2393/11/88/prepub
